# In Situ Formation of Y_2_Si_2_O_7_–Corundum–Mullite Ceramic Composites with Enhanced Thermal Shock Resistance

**DOI:** 10.3390/ma19081628

**Published:** 2026-04-18

**Authors:** Wentao Wang, Jiafei Tan, Xueying Zhang, Qi Zhang, Jiachen Liu

**Affiliations:** 1 Key Lab of Advanced Ceramics and Machining Technology of Ministry of Education, School of Materials Science and Engineering, Tianjin University, Tianjin 300072, China; wang_wt0803@163.com (W.W.); zhangqlirr@163.com (Q.Z.); 2 Beijing Institute of Astronautical Systems Engineering, Beijing 100076, China; tanjf2021@hotmail.com

**Keywords:** corundum–mullite, thermal shock resistance, Y_2_Si_2_O_7_, thermal insulation tiles

## Abstract

**Highlights:**

- A Y_2_O_3_-SiC synergistic strategy enables in situ formation of Y_2_Si_2_O_7_ in corundum–mullite ceramics.
- Y_2_Si_2_O_7_ promotes densification and strengthens grain boundaries during sintering.
- The optimized sample exhibits improved strength and thermal shock resistance.
- Y_2_Si_2_O_7_ improves crack healing and thermal shock resistance after thermal cycling.
- The strategy balances strength and thermal shock resistance in multiphase ceramics.
- Provides guidance for gas turbine combustion chamber insulation materials.

**Abstract:**

The continuous drive for higher efficiency in gas turbines has led to increased combustion temperatures, making the thermal shock resistance of thermal insulation tiles a critical factor limiting performance. Corundum–mullite multiphase ceramics are widely used in such applications; however, their performance is often constrained by an inherent trade-off between mechanical strength and thermal shock resistance. In this work, a synergistic modification strategy based on rare-earth disilicate phases was developed, wherein Y_2_O_3_ and SiC were incorporated into a corundum–mullite matrix to enable in situ formation and controlled distribution of Y_2_Si_2_O_7_ via gel casting. During sintering, Y_2_Si_2_O_7_ acts as a transient liquid phase, facilitating densification and grain boundary strengthening; upon thermal shock, it migrates to fill and heal grain boundaries and microcracks, thereby significantly enhancing thermal shock resistance. The optimized sample S5, sintered at 1400 °C, exhibited a bulk density of 2.12 g/cm^3^ and a bending strength of 68.43 MPa. Notably, after 30 thermal shock cycles (air cooling from 1000 °C to RT), its bending strength increased to 79.71 MPa, corresponding to a 16.48% enhancement. This work provides an effective strategy for incorporating rare-earth disilicates into multiphase ceramics and offers valuable guidance for the development of high-performance components for gas turbines.

## 1. Introduction

Heavy-duty gas turbines are critical components in modern power generation systems, and elevating the temperature in combustion chambers is a primary strategy to enhance their thermal efficiency [1,2,3,4]. However, operation at temperatures approaching 1500 °C, combined with severe thermal cycling and thermoacoustic vibrations, imposes significant thermal shock risks on combustion chamber insulation tiles [5,6,7]. Consequently, the materials employed in these components must exhibit excellent thermal shock resistance, high-temperature mechanical strength, and corrosion resistance. Corundum–mullite multiphase ceramics are widely used in such environments due to their resistance, which arises from microcracks induced by the mismatch in thermal expansion coefficients between the corundum and mullite phases [8,9,10]. Nevertheless, their thermal shock resistance remains insufficient to meet the increasingly demanding service conditions, such as higher temperature gradients and rapid thermal cycling, and further improvement is therefore required [11,12].

Introducing functional phases is a common strategy to further enhance the thermal shock resistance of ceramics [13], and such approaches generally fall into two main categories. The first involves non-oxide ceramics such as SiC and Si_3_N_4_ [14,15], which possess excellent thermal shock resistance, high hardness, high thermal conductivity and low thermal expansion coefficients. Their high thermal conductivity helps relieve thermal stresses arising from surface-interior temperature gradients, while crack deflection and bridging mechanisms enhance fracture toughness [16]. For instance, mullite ceramics incorporating SiC retain 80.3% of their Young’s modulus after 25 thermal shock cycles [17]. Nevertheless, these reinforcing phases are prone to oxidation under cyclic thermal loading in high-temperature oxidizing environments, which often compromises their long-term performance [18,19,20,21].

The second category includes rare-earth oxides such as La_2_O_3_ and Y_2_O_3_ [22,23,24,25], which primarily improve mechanical properties and high-temperature stability by promoting densification and optimizing grain boundary structures. Y_2_O_3_-modified cordierite-mullite ceramics, for example, retained 87.66% of their compressive strength and 71.01% of their bending strength after three thermal shock cycles [26]. However, the toughening effect of such oxides remains limited, as the strength enhancement arises primarily from densification and grain boundary refinements, with minimal direct contribution to fracture toughness [27,28]. Moreover, repeated thermal shock may induce brittle crystalline phases or unfavorable glassy phases that could act as crack initiation sites [29,30]. Therefore, while these strategies offer certain improvements, more effective modification approaches are still required to further enhance the thermal shock resistance of ceramics.

Rare-earth disilicates (RE_2_Si_2_O_7_) combine high-temperature stability with tailorable thermal expansion behavior, making them promising candidates for multifunctional grain boundary phases [31,32]. Among them, Y_2_Si_2_O_7_ has attracted particular attention due to its high melting point and excellent chemical stability [33,34]. These properties have led to its extensive study as a key material in environmental barrier coatings (EBCs) for high-temperature applications [35,36]. Importantly, Y_2_Si_2_O_7_ also exhibits good chemical compatibility with corundum–mullite matrices under high-temperature conditions [37,38]. Its relatively low thermal expansion coefficient (3.5–5.0 × 10^−6^ K^−1^) generates residual compressive stress at grain boundaries upon cooling, contributing to crack-tip passivation [39]. In addition, its high-temperature stability helps suppress mullite decomposition, thereby stabilizing the reinforcing skeleton during thermal shock [40]. The layered crystal structure further facilitates ion migration at elevated temperatures, enabling diffusion and accumulation at grain boundaries or microcracks under thermal stress, which may promote crack filling and bridging and potentially enable dynamic crack healing [41]. These characteristics suggest that Y_2_Si_2_O_7_ may be beneficial for improving the thermal shock resistance of corundum–mullite ceramics. However, the specific role of Y_2_Si_2_O_7_ in this context has received relatively limited attention, and its toughening mechanisms under severe thermal shock conditions remain poorly understood. Therefore, clarifying the migration behavior of Y_2_Si_2_O_7_ under thermal stress and its contribution to thermal shock resistance is essential.

In this study, Y_2_Si_2_O_7_ was synthesized in situ within a corundum–mullite matrix by synergistically introducing Y_2_O_3_ and SiC via gel casting, enabling the fabrication of complex-shaped insulation tiles. The effects of Y_2_Si_2_O_7_ on phase composition, microstructure, mechanical properties, and thermal shock resistance were systematically investigated, with particular emphasis on its migration behavior during sintering and thermal shock and the associated toughening mechanisms. A corundum–mullite multiphase ceramic exhibiting enhanced strength, fracture toughness, and superior thermal shock resistance was successfully developed through the controlled synthesis and regulation of Y_2_Si_2_O_7_.

## 2. Experimental Methods

### 2.1. Raw Materials

Al_2_O_3_ (1 μm, Guangzhou Metal & Metallurgy Co., Ltd., Guangzhou, China), kaolin (11 μm, Tianjin Zhiyuan Chemical Reagent Co., Ltd., Tianjin, China), and tabular corundum (45 μm, Almatis Aluminum Co., Ltd., Qingdao, China) were used as the main raw materials. Y_2_O_3_ and SiC (purity > 99%, Aladdin Reagent Co., Ltd., Shanghai, China) were employed as additives. Acrylamide (AM, >99%), N,N′-methylenebisacrylamide (MBAM, >99%), ammonium polyacrylate (PAA-NH4), ammonium persulfate (APS, ≥98%) and N,N,N′,N′-tetramethylethylenediamine (TEMED, >99%) were purchased from Aladdin Reagent Co., Ltd., Shanghai, China. The compositions of samples S1–S6 are summarized in Table 1. All component ratios are expressed in weight percentage (wt.%). Sample S1 was designed as the baseline without additives to represent the intrinsic behavior of the corundum–mullite matrix, while samples S2–S6 were systematically varied to evaluate the individual and synergistic effects of SiC and Y_2_O_3_.

### 2.2. Sample Preparation

**Figure 1:**
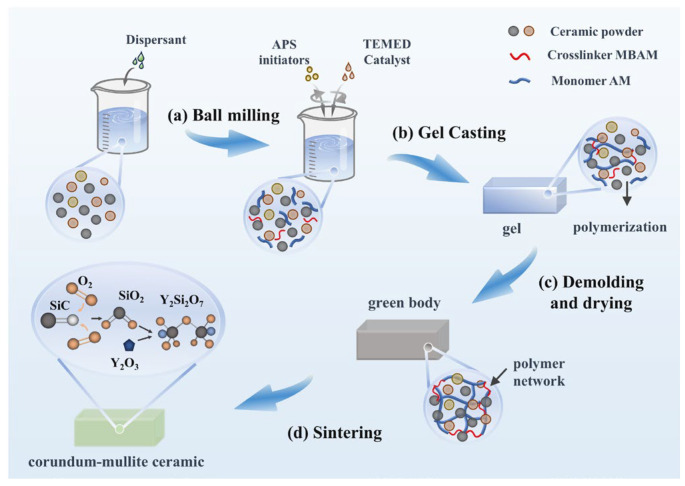
Schematic diagram of sample preparation process.

A water-based gel-casting process was used to prepare the samples in this study. According to the designed S-series formulation, raw materials were weighed and processed following the procedure illustrated in Figure 1. Initially, ceramic powders were uniformly mixed with the dispersant PAA-NH_4_ and deionized water; the dispersant concentration was 0.6 wt.% and the slurry solid content was 80 wt.%. The mixture was then ball-milled at 300 rpm for 2 h with a ball-to-slurry ratio of 1:1 (by mass). Subsequently, the monomer (AM) and crosslinking agent (MBAM) were introduced into the ball-milled slurry and homogenized. Thereafter, the catalyst (TEMED) and initiator (APS) were added to initiate gelation for shaping. After demolding, the green bodies were dried and then sintered in a muffle furnace to obtain the final ceramic samples. The prepared samples’ dimensions were as follows: (1) Strip-shaped samples measuring 5 × 10 × 50 mm; (2) Strip-shaped samples measuring 3 × 4 × 35 mm. These samples were sintered at 1200–1500 °C with a heating rate of 5 °C/min, and held at the peak temperature for 2 h. The thermal shock resistance of the sintered samples was evaluated using a muffle furnace. The thermal shock test was conducted as follows: the sintered samples were heated in a muffle furnace at 5 °C/min to 1000 °C and held for 15 min, followed by air-cooling to ambient temperature. The selected temperature of 1000 °C provides a sufficiently large temperature gradient (ΔT) to induce significant thermal stress and crack evolution. Although the combustor gas temperature can reach approximately 1500 °C, the combustion chamber liner is protected by ceramic insulation tiles and backside cooling, resulting in a much lower surface temperature of the ceramic components, typically ranging from several hundred to approximately 1000 °C [42]. More importantly, thermal shock damage is primarily governed by the temperature difference rather than the absolute temperature. During transient processes such as shutdown, the temperature drop is approximately 1000 °C, leading to severe thermal stresses [43]. Therefore, a thermal shock test between 1000 °C and room temperature corresponds to a temperature difference of approximately 1000 °C, which is representative of practical service conditions. In addition, such testing conditions are widely adopted for evaluating the cyclic thermal shock resistance of oxide ceramics [24,44,45,46,47,48,49,50]. This procedure constituted one thermal shock cycle. After *n* thermal shock cycles (*n* = 5, 10, 15, 20, 25, 30), the bending strength of the samples was measured. The residual bending strength was used to assess their thermal shock resistance.

### 2.3. Characterization

The water absorption rate (Wa) and open porosity (P) were determined using the principle of Archimedes. Linear shrinkages of samples were calculated in accordance with Equation (1).

(1) $$L=\frac{l_{0}-l_{1}}{l_{0}}\times 100\%,$$

where *L* is the linear shrinkage, *l*_0_ is the original length, and *l*_1_ is the length after sintering.

The sintered samples were subjected to X-ray diffraction (XRD, D8 advance, Bruker, Karlsruhe, Germany) examinations within the 2θ range of 10–80° at a scanning rate of 2°/min (step size 0.02°) to identify their phase composition. The microstructure of the materials was analyzed using field emission scanning electron microscopy (Hitachi SU8600, Hitachi High-Tech Corporation, Tokyo, Japan) and an energy dispersive spectrometer (EDS, 7401 Oxford, Oxford Instruments, Concord, MA, USA), and the elemental distribution in the samples was obtained concurrently. A thermal analyzer (Hot Disk TPS 2500s, Gothenburg, Sweden) was used to measure the sintered samples’ thermal conductivity at room temperature. The samples’ coefficient of thermal expansion (CTE) was measured using a high-temperature thermal expansion meter (DIL 402CL, NETZSCH, Selb, Germany) throughout a temperature range of 25–1000 °C. Bending strength (σ) and fracture toughness (K_IC_) were measured with a universal testing equipment (CMT4304, MTS Systems Corporation, Eden Prairie, MN, USA). The three-point bending method was used to test the bending strength of the samples on test strips that measured 5 × 10 × 50 mm. The loading rate was 0.05 mm/min and the support spacing was 20 mm. The reported value was the average of five measurements. Equation (2) was used to calculate the bending strength value.

(2) $$\sigma =\frac{3PL}{2bh^{2}},$$

where *P* is the fracturing load, *L* is span, *b* is the sample width and *h* is the sample thickness.

The single-sided notched beam (SENB) method was used to measure fracture toughness (*K_IC_*) in the three-point bending mode. The rectangular test strips utilized were 3 × 4 × 35 mm, with support spacing and loading rate of 24 mm and 0.05 mm/min, respectively. The test strips had a cut depth of roughly 2 mm and a width of roughly 0.2 mm. Each reported value represents the average of five independent measurements to ensure reproducibility. Equations (3) and (4) were used to determine the specimen’s fracture toughness (*K_IC_*). Here, *f*($\frac{a}{h}$) is defined as the geometry factor, which is a function of the relative crack length (*a*/*h*), as given in Equation (4).

(3) $$K_{IC}=\frac{PL}{bh^{\frac{3}{2}}}\times f\left(\frac{a}{h}\right),$$

(4) $$f\left(\frac{a}{h}\right)=2.9{\left(\frac{a}{h}\right)}^{\frac{1}{2}}-4.6{\left(\frac{a}{h}\right)}^{\frac{3}{2}}+21.8{\left(\frac{a}{h}\right)}^{\frac{5}{2}}-37.6{\left(\frac{a}{h}\right)}^{\frac{7}{2}}+38.7{\left(\frac{a}{h}\right)}^{\frac{9}{2}},$$

where *a* stands for the crack depth that was inserted into the sample, and the other symbols have the same meanings as those in Equation (2).

## 3. Results and Discussion

### 3.1. Physical Properties of the Samples

**Figure 2:**
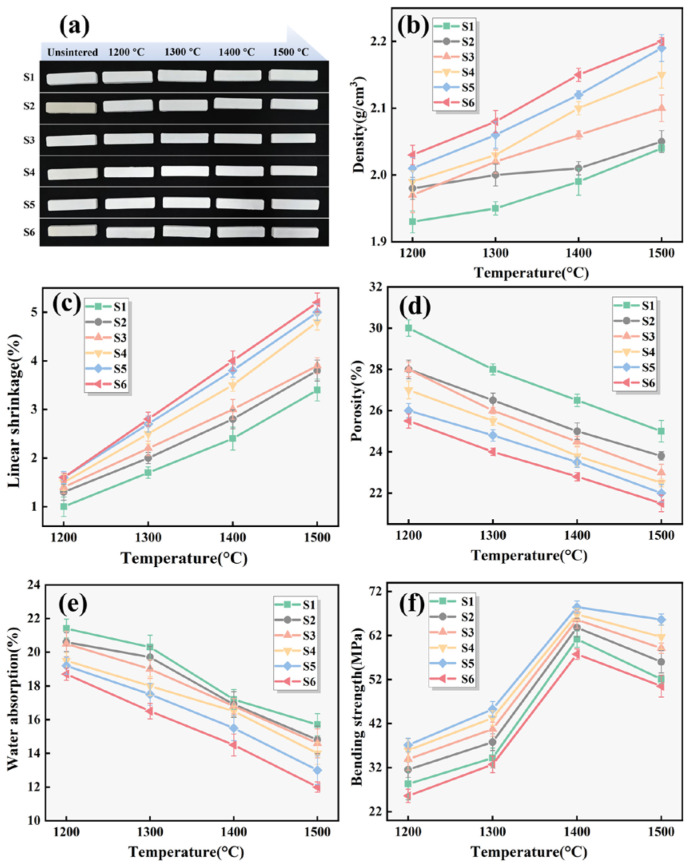
Relationships between the sintering temperature and (**a**) macroscopic morphology, (**b**) bulk density, (**c**) linear shrinkage, (**d**) porosity, (**e**) water absorption and (**f**) bending strength of the samples.

Figure 2 presents the macroscopic morphology of the samples before and after sintering at different temperatures, together with the variations in bulk density, linear shrinkage, water absorption, porosity, and bending strength. With increasing sintering temperature (1200–1500 °C), porosity and water absorption decreased, whereas bulk density and linear shrinkage increased progressively. Compared with S1 (without additives), the introduction of Y_2_O_3_ and SiC significantly improved densification. After sintering at 1500 °C, S1 exhibited a density of 2.04 g/cm^3^ and a porosity of 25%, whereas S2–S6 showed improved properties, suggesting that the addition of Y_2_O_3_ and SiC contributes to liquid-phase-assisted densification. Figure 2f shows the variation in bending strength with temperature. The bending strength increased from 1200 to 1400 °C for all samples and then declined at 1500 °C. S2 showed higher strength than S1, which is likely associated with the formation of a liquid phase induced by SiC oxidation, enhancing sintering and mechanical performance. The bending strength of S3 was lower than that of S4, suggesting that the presence of SiC may facilitate the transformation of Y_2_O_3_ into Y_2_Si_2_O_7_, thereby further improving strength. S5 (3% Y_2_O_3_, 1.07% SiC) achieved the maximum bending strength of 68.43 MPa at 1400 °C, whereas excessive SiC in S6 reduced strength to 57.76 MPa, possibly due to the formation of excessive grain boundary phase and local embrittlement. The decline in strength for all samples at 1500 °C may be related to abnormal grain growth and over-sintering effects. Overall, 1400 °C was identified as the optimal sintering temperature.

### 3.2. Analysis of Phase Composition

**Figure 3:**
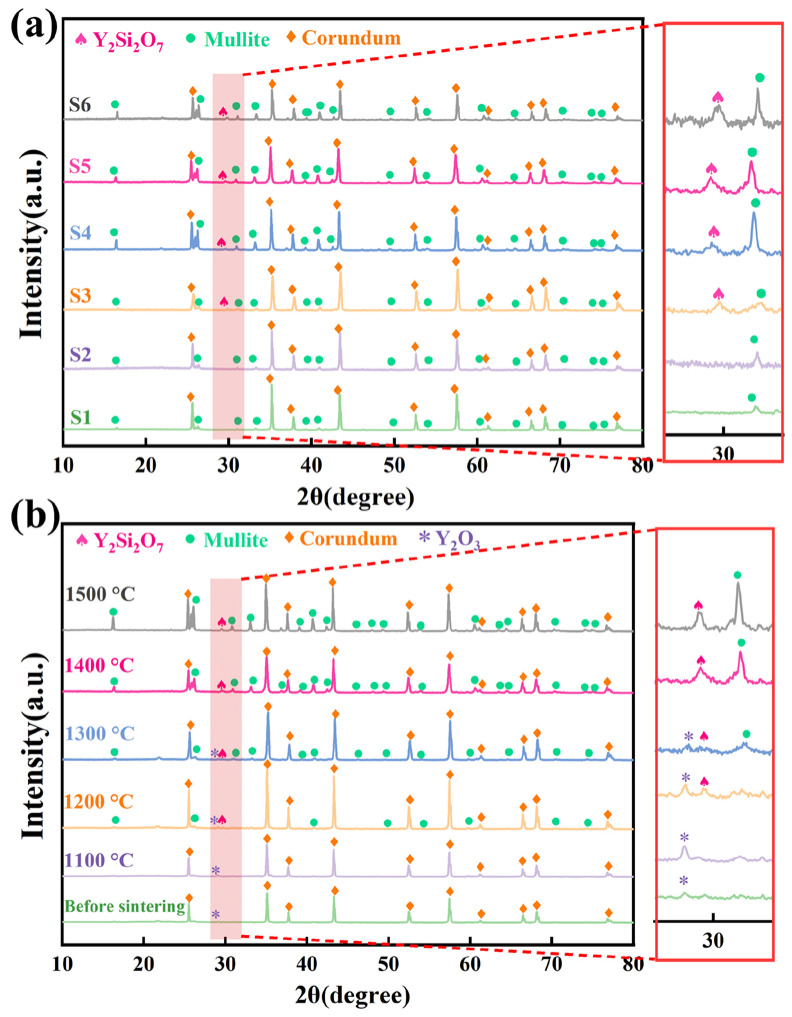
(**a**) XRD patterns of S1–S6 samples sintered at 1400 °C, (**b**) XRD patterns of S5 sample before sintering and after sintering at different temperatures.

Figure 3a illustrates the XRD patterns of samples S1–S6 after sintering at 1400 °C. The major crystalline phase of samples S1–S6 was corundum, with mullite as the secondary crystalline phase. The S2 sample with 1.07% SiC added alone still exhibited only two phases, corundum and mullite, indicating that SiC was oxidized to SiO_2_ during the sintering process (Equation (5)) and then reacted with Al_2_O_3_ in the matrix to generate the mullite phase (Equation (6)).

(5) $$SiC+\frac{3}{2}O_{2}\to SiO_{2}+CO,$$

(6) $$2SiO_{2}+3{Al}_{2}O_{3}\to 3{Al}_{2}O_{3}\cdot 2SiO_{2}\left(mullite\right),$$

(7) $$Y_{2}O_{3}+2SiO_{2}\to Y_{2}{Si}_{2}O_{7},$$

The S3 sample (1.5% Y_2_O_3_ only) exhibited characteristic Y_2_Si_2_O_7_ diffraction peaks at 2θ = 29.5°, indicating that Y_2_O_3_ can react with SiO_2_ present in the ceramic matrix to form Y_2_Si_2_O_7_ (Equation (7)), though the peak intensity was weaker than those in S4–S6. In S4–S6, the intensity of the Y_2_Si_2_O_7_ peak increased with increasing additions of SiC and Y_2_O_3_, indicating that the oxidation of SiC generates highly reactive in situ SiO_2_, which reacts with Y_2_O_3_ to promote the formation of Y_2_Si_2_O_7_.

To clarify the formation temperature and evolution of Y_2_Si_2_O_7_, XRD analysis was conducted on S5 before and after sintering at different temperatures (Figure 3b). In the green body, corundum was the dominant crystalline phase, and a characteristic Y_2_O_3_ peak was detected at 2θ = 29.16°. After sintering at 1100 °C, only corundum and Y_2_O_3_ were present, while mullite had not yet formed. At 1200 °C, mullite and Y_2_Si_2_O_7_ peaks appeared simultaneously, indicating an initial formation temperature of approximately 1200 °C, with residual Y_2_O_3_ suggesting incomplete reaction. At 1300 °C, Y_2_O_3_ coexisted with Y_2_Si_2_O_7_, and above 1400 °C, Y_2_O_3_ was nearly completely converted into Y_2_Si_2_O_7_, indicating that the reaction in Equation (7) progressed mainly between 1300 and 1400 °C.

Compared with S1, the mullite diffraction peaks in S4–S6 are significantly intensified, indicating enhanced mullite formation in the presence of SiC. Although mullite (Equation (6)) and Y_2_Si_2_O_7_ (Equation (7)) both consume SiO_2_ and may therefore appear to be competing reactions, the experimental results indicate that mullite formation is not suppressed in this system. This behavior can be attributed to synergistic thermodynamic and kinetic effects rather than a direct increase in SiO_2_ availability. From a thermodynamic standpoint, the introduction of Y_2_O_3_ facilitates the formation of liquid phases represented by Y_2_Si_2_O_7_ at relatively lower temperatures. These liquid phases enhance mass transport and may reduce the effective reaction barrier for mullite formation, thereby promoting mullite crystallization over a broader temperature range [51,52,53]. From a kinetic perspective, the presence of such liquid phases decreases the viscosity of the grain boundary region and enhances ionic diffusion (e.g., Al^3+^ and Si^4+^), thereby accelerating mullite nucleation and growth [54]. Meanwhile, the oxidation of SiC provides the SiO_2_ required for the formation of Y_2_Si_2_O_7_ phases. In order to prevent SiO_2_ consumption for Y_2_Si_2_O_7_ creation from competing with mullite formation, a suitable quantity of SiC is added in this study to guarantee its complete interaction with Y_2_O_3_. As a result of these combined effects, enhanced mullite formation is achieved despite the nominal competition for SiO_2_. Therefore, this enhancement should be understood as a synergistic thermodynamic-kinetic facilitation associated with Y_2_Si_2_O_7_-related liquid phases, rather than a direct chemical driving force. Furthermore, Y_2_Si_2_O_7_ exhibits high chemical stability below 1600 °C [40], allowing it to persist as a stable grain boundary phase during sintering and contribute to microstructural stability.

### 3.3. Micro-Morphological Analysis of the Samples

**Figure 4:**
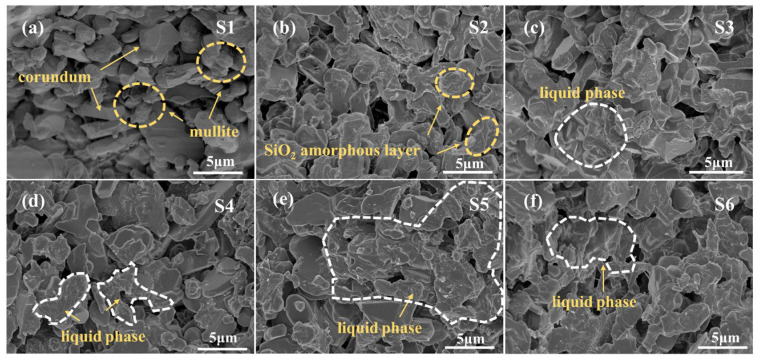
SEM images of the fracture morphology of samples after sintering at 1400 °C: (**a**) S1; (**b**) S2; (**c**) S3; (**d**) S4; (**e**) S5; (**f**) S6.

Figure 4 presents SEM images of the fracture surfaces of S1–S6 sintered at 1400 °C. Sample S1 exhibits a typical corundum–mullite two-phase structure, where corundum particles are encapsulated and interconnected by columnar mullite grains. Clear grain boundaries and numerous irregular pores are observed, mainly located at grain boundary junctions. In S2, an amorphous SiO_2_ layer formed by the oxidation of SiC is observed. The additional SiO_2_ promotes further mullite formation. Compared with S1, S3 exhibits significantly reduced porosity, attributed to the densification effect of Y_2_O_3_. A Y_2_Si_2_O_7_ liquid phase forms thin films along grain boundaries, contributing to enhanced bending strength. With the combined addition of Y_2_O_3_ and SiC, S4–S6 display a more continuous liquid-phase distribution at grain boundaries and a higher mullite content relative to S1. In S4, the amorphous Y_2_Si_2_O_7_ phase appears as isolated and discontinuous regions along grain boundaries. In S6, excessive oxidation of SiC produces abundant SiO_2_, which reacts with Y_2_O_3_, forming thick liquid films or aggregates covering grain surfaces, resulting in local microstructural inhomogeneity and strength deterioration. In contrast, S5 exhibits a relatively dense and homogeneous fracture morphology, with a uniformly distributed thin liquid film along grain boundaries, leading to an optimized microstructure. Consistent with the results shown in Figure 2, S5 exhibits the best overall properties.

**Figure 5:**
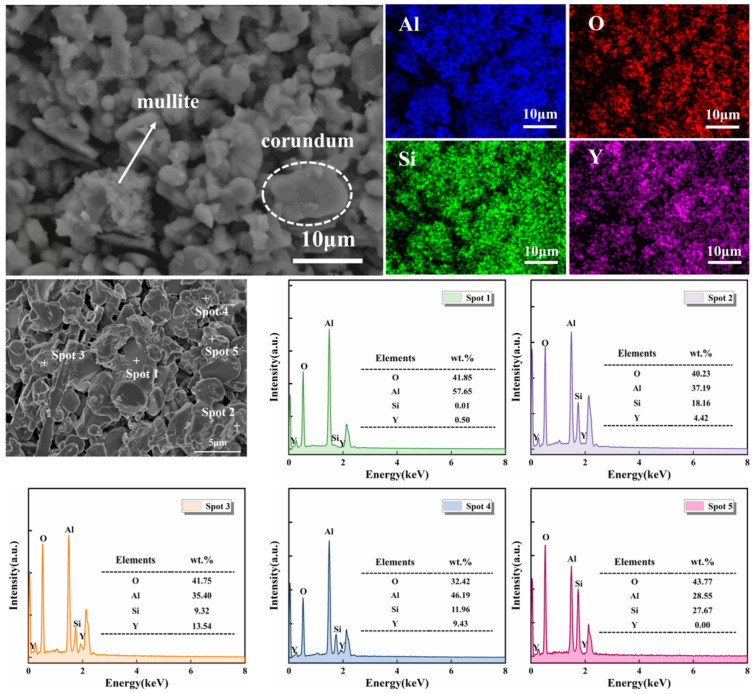
Element mapping images and EDS spectra of selected spots of S5 after sintering at 1400 °C.

**Figure 6:**
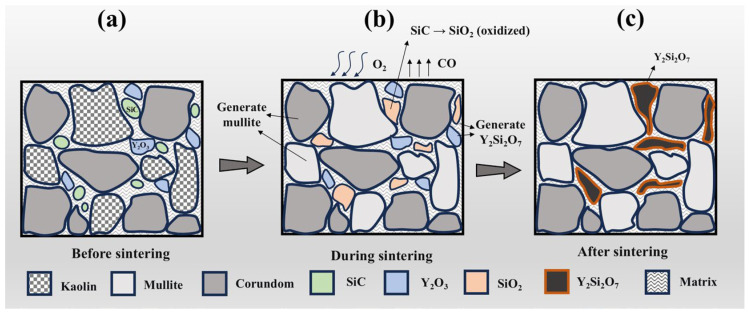
Schematic illustration of the synergistic reaction between SiC and Y_2_O_3_ during sintering: (**a**) before sintering; (**b**) during sintering; (**c**) after sintering.

To verify the phase composition and microstructure, EDS analysis was conducted on the S5 sample sintered at 1400 °C. As shown in Figure 5, regions rich in Al and O correspond to corundum, the dominant phase in this area, while regions enriched in Al, O, and Si represent mullite. Y is discontinuously distributed along grain boundaries, partially overlapping with Si, indicating the formation of Y_2_Si_2_O_7_ without complete coverage. Spot EDS analysis shows that the Al:O stoichiometry at spot 1 matches corundum. Spot 2 corresponds to columnar mullite with elevated Y, suggesting a mixed mullite/Y_2_Si_2_O_7_ region. Spot 3 (Y:Si ≈ 1.45) is consistent with Y_2_Si_2_O_7_ enrichment, and spot 4 (Y:Si ≈ 0.79) represents a reaction interface. Spot 5 contains high Si and no Y, likely residual amorphous SiO_2_ from SiC oxidation. Y_2_Si_2_O_7_ is primarily located at grain boundaries, forming clear interfaces with the corundum–mullite matrix, with Y content increasing from the grain interior to the boundaries, indicating preferential precipitation at high-energy sites. The adjacency of Si-rich regions to Y-rich boundaries confirms that SiO_2_ from SiC oxidation reacts with Y_2_O_3_ to form Y_2_Si_2_O_7_. Combined with XRD and SEM results, the synergistic reaction mechanism between SiC and Y_2_O_3_ is schematically illustrated in Figure 6.

### 3.4. Mechanical Property Analysis of the Samples

**Figure 7:**
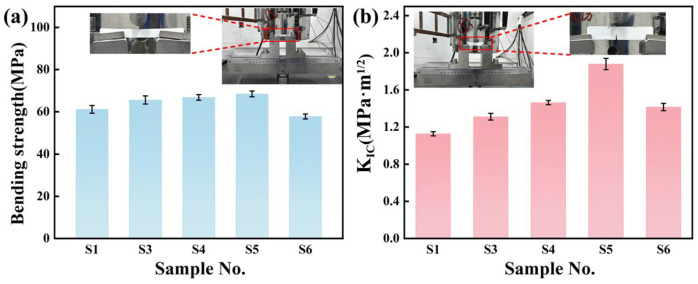
(**a**) Bending strength and (**b**) fracture toughness of the samples sintered at 1400 °C.

The bending strength and fracture toughness (K_IC_) of samples sintered at 1400 °C are presented in Figure 7a,b. As shown in Figure 7, S1 exhibits a bending strength of 61.14 MPa and a fracture toughness of 1.13 MPa·m^1/2^. The limited densification resulting from pure solid-state sintering and the absence of grain boundary reinforcing phases allow cracks to propagate readily along grains, resulting in low toughness. S3 exhibited an increased bending strength of 65.58 MPa compared with S1, while its fracture toughness is 1.31 MPa·m^1/2^, lower than that of S4–S6. This indicates that Y_2_Si_2_O_7_ contributes to the enhancement of both bending strength and fracture toughness. S5 shows the highest bending strength (68.43 MPa), with a fracture toughness of 1.88 MPa·m^1/2^. In S5, the added Y_2_O_3_ reacts with SiO_2_ generated from SiC oxidation to form a sufficient in situ Y_2_Si_2_O_7_ grain boundary phase. The amorphous SiO_2_ interfacial layer between Y_2_Si_2_O_7_ and the matrix facilitates microcrack initiation, promoting crack deflection and bifurcation. Moreover, the coefficient of thermal expansion (CTE) of amorphous SiO_2_ (0.5 × 10^−6^ K^−1^) is much lower than that of Y_2_Si_2_O_7_ (3.5–5 × 10^−6^ K^−1^) [39]. This CTE mismatch generates local compressive stresses, increasing the energy barrier for crack propagation and thereby enhancing toughness. Comparison of S4–S6 indicates that the Y_2_Si_2_O_7_ content in S5 is optimal, enabling the formation of a continuous grain boundary phase and preventing the embrittlement caused by excessive Y_2_Si_2_O_7_ in S6.

### 3.5. Thermal Shock Resistance of the Samples

**Figure 8:**
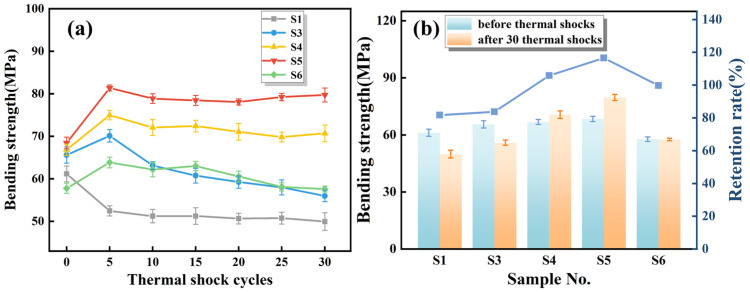
Bending strength of samples sintered at 1400 °C after thermal shock: (**a**) variation of bending strength as a function of thermal shock cycles; (**b**) bending strength before and after 30 cycles with retention rate.

During gas turbine operation, the combustion chamber undergoes repeated extreme heating and cooling; therefore, insulation tiles require excellent thermal shock resistance for long-term service. To evaluate the effects of SiC and Y_2_O_3_ additions, samples S1–S6 sintered at 1400 °C were subjected to 30 thermal shock cycles (room temperature—1000 °C). Figure 8 presents the relationship between bending strength and the number of thermal shock cycles. As shown in Figure 8a, the bending strength of S1 gradually decreases with increasing cycle number. S1, consisting of a corundum–mullite matrix, lacks grain boundary repair capability, allowing cracks to propagate rapidly once initiated. In contrast, S3–S6 exhibit a slight strength increase after five cycles, followed by a gradual decline. Notably, the strength reduction in S4–S6 (with both SiC and Y_2_O_3_) is significantly smaller than in S3 (Y_2_O_3_ only). XRD results indicate that the addition of SiC promotes the formation of the Y_2_Si_2_O_7_ phase, which plays an important role in improving the thermal shock resistance of the ceramics. Without a sufficiently developed Y_2_Si_2_O_7_ grain boundary network, crack healing and mullite stabilization during cyclic thermal shocks are limited, resulting in faster degradation of mechanical properties. As shown in Figure 8b, S5 exhibits the best thermal shock resistance among all samples. After 30 thermal shock cycles, its bending strength remains 79.71 MPa, corresponding to a strength retention of 116.48%. This behavior is consistent with the previously discussed fracture toughness and phase evolution results. The optimized SiC:Y_2_O_3_ ratio in S5 promotes the formation of a continuous Y_2_Si_2_O_7_ grain boundary network while maintaining the stability of the mullite phase. Such a microstructure effectively deflects and arrests crack propagation during thermal shock, thereby significantly enhancing resistance to repeated thermal shocks between 1000 °C and room temperature.

**Figure 9:**
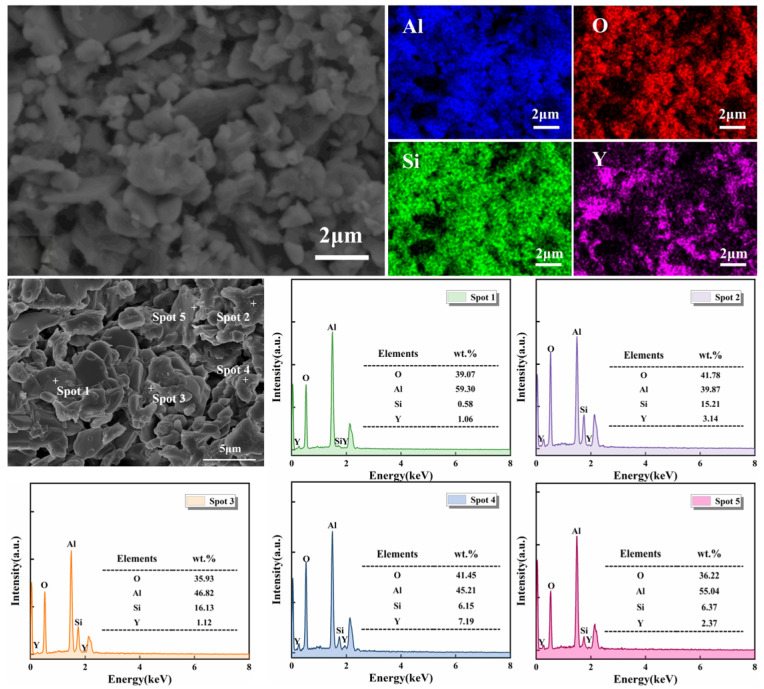
Element mapping images and EDS spectra of selected spots of S5 sintered at 1400 °C after 30 thermal shock cycles.

To further elucidate the microstructural evolution of S5 after 30 thermal shock cycles, EDS analysis was performed, and the results are shown in Figure 9. Compared with the state prior to thermal shock, the Y_2_Si_2_O_7_ grain boundary layer becomes noticeably thicker after 30 cycles. The Y signal intensity at grain boundaries increases and its distribution becomes more continuous. Similarly, the Si signal at grain boundaries is enhanced, and its co-localization region with Y expands, suggesting an increased presence and redistribution of the Y_2_Si_2_O_7_ phase after repeated thermal shocks. Local compositional analysis of the selected spots indicates that spot 1 mainly corresponds to corundum, while spot 3 corresponds to mullite. At spot 2, the Al:Si ratio of approximately 2.6:1 is close to that of mullite, but the relatively high Y content suggests a mixed mullite/Y_2_Si_2_O_7_ region, indicating preferential precipitation of Y_2_Si_2_O_7_ in Si-rich areas. At spot 4, a Y:Si ratio of approximately 1.17 approaches the theoretical value of Y_2_Si_2_O_7_, indicating a Y_2_Si_2_O_7_-enriched region. Compared with the corresponding region in Figure 5, the Y:Si ratio is closer to the ideal stoichiometric proportion, which suggests that residual Y_2_O_3_ is further consumed and that the formation of Y_2_Si_2_O_7_ becomes more complete after thermal shock cycling. Spot 5, characterized by high Al and low Si and Y contents, mainly corresponds to corundum with a small amount of Y_2_Si_2_O_7_ at the grain boundary. It should be noted that although Y_2_Si_2_O_7_ is formed during the sintering process, its primary role at this stage is associated with liquid-phase-assisted densification, where the liquid phase facilitates mass transport and pore filling under near-equilibrium conditions. In contrast, during thermal shock cycling, cyclic thermal stresses and temperature gradients induce the formation of microcracks and high-energy defect sites. Under these non-equilibrium conditions, the grain boundary phase can locally redistribute and flow into microcrack regions, resulting in crack filling and defect accommodation. Therefore, the underlying mechanism is essentially consistent, as both processes are governed by liquid-phase flow and filling while the observed effect differs due to the nature of the defects involved, namely pore elimination during sintering and microcrack healing during thermal shock.

After 30 thermal shock cycles, the enrichment and redistribution of Y_2_Si_2_O_7_ at grain boundaries become more pronounced. Thermal stresses and temperature gradients promote the redistribution of Y-containing species from grain interiors toward grain boundaries and adjacent crack regions, leading to the enrichment of Y_2_Si_2_O_7_ in these areas. This process facilitates the partial filling of microcracks and grain boundary defects, thereby reducing stress concentration and inhibiting crack propagation. The continuous distribution of Y_2_Si_2_O_7_ along grain boundaries enhances intergranular bonding and suppresses thermally induced intergranular fracture, which contributes to the observed increase in bending strength of S5 after thermal shock cycling.

**Figure 10:**
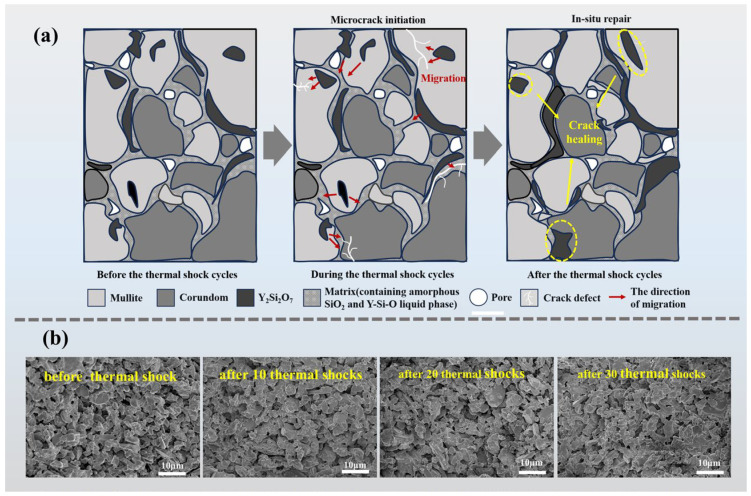
(**a**) Schematic illustration of the strength improvement mechanism of sample S5 after 30 thermal shock cycles; (**b**) SEM images of sample S5 before thermal shock and after different numbers of thermal shock cycles.

Figure 10a schematically illustrates the strengthening mechanism. During repeated thermal cycling, thermal stresses promote the diffusion, migration, and enrichment of the Y_2_Si_2_O_7_ grain boundary phase toward high energy regions such as microcracks and grain boundary defects. This redistribution facilitates defect filling and inhibits crack propagation, thereby enhancing grain boundary cohesion and improving strength. SEM images in Figure 10b further show that after several thermal shock cycles, the grain boundary phase becomes more uniformly distributed, while the overall microstructure remains stable, indicating excellent thermal shock resistance. This observation correlates with the bending strength evolution in Figure 8 and provides microstructural evidence that thermally driven migration and enrichment of Y_2_Si_2_O_7_ contribute to grain boundary strengthening and improved mechanical performance.

### 3.6. Thermophysical Properties of the Samples

**Figure 11:**
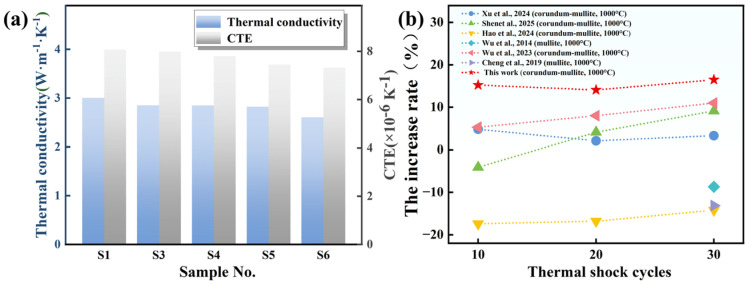
(**a**) Thermal conductivity and coefficient of thermal expansion of samples sintered at 1400 °C; (**b**) strength growth rate after thermal shocks reported in different studies [45,46,47,48,49,50].

To further clarify the relationship between thermophysical properties and thermal shock resistance, the thermal conductivity and coefficient of thermal expansion (CTE) of samples sintered at 1400 °C were measured, as shown in Figure 11a. Thermal shock resistance is generally governed by a combination of thermophysical and mechanical properties, among which thermal conductivity and CTE play particularly important roles. A lower CTE can reduce thermal stress induced by temperature gradients, while higher thermal conductivity facilitates heat dissipation and alleviates internal temperature differences within the material [55]. Comparison of the thermal conductivity of samples shows that S1 exhibits the highest value of 3.00 W·m^−1^·K^−1^. Heat transport in ceramics is primarily governed by lattice vibrations in the form of phonons [44]. As the Y_2_Si_2_O_7_ content increases from S3 to S6, thermal conductivity gradually decreases. This is because the Y_2_Si_2_O_7_ phase has relatively low thermal conductivity (approximately 1.5–3 W·m^−1^·K^−1^) and forms a continuous network along grain boundaries, acting as an effective phonon-scattering center, thereby reducing overall thermal conductivity. Meanwhile, the CTE of the samples also decreases with increasing Y_2_Si_2_O_7_ content. On the one hand, SiC undergoes oxidation during sintering to produce amorphous SiO_2_, which is more reactive than the original SiO_2_ in the raw materials. This reactive SiO_2_ readily reacts with Al_2_O_3_, significantly promoting the formation and growth of mullite. The CTE of mullite (approximately 5.0–5.5 × 10^−6^ K^−1^) is substantially lower than that of corundum (approximately 8.0–8.5 × 10^−6^ K^−1^) [9], so an increased volume fraction of mullite reduces the overall CTE. On the other hand, residual amorphous SiO_2_ (CTE ≈ 0.5 × 10^−6^ K^−1^) and the formed Y_2_Si_2_O_7_ phase (CTE 3.5–5.0 × 10^−6^ K^−1^) generate a gradient CTE mismatch with the corundum–mullite matrix, further contributing to the reduction in the overall CTE.

Based on these results, a qualitative relationship can be established in which a combination of relatively high thermal conductivity and low CTE is favorable for thermal shock resistance. Among the samples, S5 best satisfies this balance. Although S6 exhibits a lower CTE, its thermal conductivity decreases significantly, while S1 maintains a relatively high thermal conductivity but does not achieve sufficient CTE reduction. It should be emphasized that the evaluation of thermal shock resistance in this study is primarily based on experimental thermal shock results, and the above discussion is intended to provide a qualitative interpretation of the observed behavior based on measured thermophysical properties.

Combined with the previous analysis of thermal shock resistance, it is evident that the performance of the samples is not governed solely by thermophysical parameters. As shown in Figure 11b, most reported oxide-based ceramic systems exhibit strength variations ranging approximately from −17.4% to 11% after thermal shock cycling [45,46,47,48,49,50]. In contrast, the present material (S5) shows a significantly higher strength increase of 16.48%, representing the highest strength improvement among the compared systems and demonstrating superior thermal shock resistance. In this system, the Y_2_Si_2_O_7_ phase is considered to play an important role in enhancing thermal shock resistance in S5. The oxidation of SiC generates SiO_2_, which reacts with the added Y_2_O_3_ to form a suitably proportioned and relatively uniformly distributed Y_2_Si_2_O_7_ grain boundary phase. This continuous Y_2_Si_2_O_7_ network is suggested to contribute to the stabilization of the mullite phase at high temperatures and to help maintain the structural integrity of the matrix during thermal cycling. Moreover, under thermal stress, Y_2_Si_2_O_7_ is believed to redistribute toward grain boundaries and microcrack regions, contributing to defect filling and crack-tip blunting. These mechanisms collectively improve resistance to crack initiation and growth, allowing S5 to retain a 16.48% increase in strength after 30 thermal shock cycles, significantly outperforming the other samples.

## 4. Conclusions

In this study, corundum–mullite ceramics were successfully prepared via gel casting, and the effects of Y_2_Si_2_O_7_ content on phase composition, microstructure, and properties were systematically investigated. Among the samples, S5, containing 3 wt.% Y_2_O_3_ and 1.07 wt.% SiC, exhibited the best overall performance after sintering at 1400 °C, with a bending strength of 68.43 MPa, fracture toughness of 1.88 MPa·m^1/2^, bulk density of 2.12 g/cm^3^, a CTE of 7.45 × 10^−6^ K^−1^ (25–1000 °C), and a thermal conductivity of 2.83 W·m^−1^·K^−1^. Microstructural analysis revealed that the oxidation of SiC generates highly reactive SiO_2_, which reacts with Y_2_O_3_ to form Y_2_Si_2_O_7_ in situ along grain boundaries. Y_2_Si_2_O_7_ promotes densification and strengthens grain boundary bonding, while under thermal shock, it migrates toward microcracks, filling defects and facilitating crack healing. These synergistic effects of Y_2_Si_2_O_7_ formation and migration enable S5 to retain a 16.48% increase in strength after 30 severe thermal shock cycles between room temperature and 1000 °C. Overall, Y_2_Si_2_O_7_ not only enhances the material’s strength but also establishes a dynamic repair network, which is critical to the exceptional thermal shock resistance of these multiphase ceramics.

## Figures and Tables

**Table 1:** Compositions of samples S1–S6 (wt.%).

Sample	Al_2_O_3_ (wt.%)	Kaolin (wt.%)	Tabular Corundum (wt.%)	Y_2_O_3_ (wt.%)	SiC (wt.%)
S1	80	10	10	0	0
S2	80	10	10	0	1.07
S3	80	10	10	1.5	0
S4	80	10	10	1.5	0.53
S5	80	10	10	3	1.07
S6	80	10	10	6	2.13

## Data Availability

The original contributions presented in this study are included in the article. Further inquiries can be directed to the corresponding authors.

## Abbreviations

The following abbreviations are used in this manuscript:

CTE	Coefficient of Thermal Expansion
RT	Room Temperature
